# Prevalence and predictors of fatigue after aneurysmal subarachnoid hemorrhage

**DOI:** 10.1007/s00701-020-04538-9

**Published:** 2020-08-18

**Authors:** Elin Western, Angelika Sorteberg, Cathrine Brunborg, Tonje Haug Nordenmark

**Affiliations:** 1 Department of Neurosurgery, Oslo University Hospital, P O Box 4950, Nydalen, 0424 Oslo, Norway; 2 Faculty of Medicine, Institute of Clinical Medicine, University of Oslo, Oslo, Norway; 3 Oslo Centre for Biostatistics and Epidemiology, Research Support Services, Oslo University Hospital, Oslo, Norway; 4 Department of Physical Medicine and Rehabilitation, Oslo University Hospital, Oslo, Norway; 5 Department of Psychology, University of Oslo, Oslo, Norway

**Keywords:** Aneurysmal subarachnoid hemorrhage, SAH, Fatigue, Prevalence, Predictors

## Abstract

**Background:**

Fatigue is a common and disabling sequel after aneurysmal subarachnoid hemorrhage (aSAH). At present, prevalence estimates of post-aSAH fatigue in the chronic phase are scarce and vary greatly. Factors from the acute phase of aSAH have hitherto barely been associated with post-aSAH fatigue in the chronic phase.

**Methods:**

Prospective study assessing prevalence of fatigue using the Fatigue Severity Scale (FSS) in patients who were living independently 1 to 7 years after aSAH. We compared demographic, medical, and radiological variables from the acute phase of aSAH between patients with and without fatigue (FSS ≥ 4 versus < 4) and searched for predictors of fatigue among these variables applying univariable and multivariable regression analyses.

**Results:**

Of 726 patients treated for aSAH in the period between January 2012 and December 2017, 356 patients completed the assessment. The mean FSS score was 4.7 ± 1.7, and fatigue was present in 69.7%. The frequency of patients with fatigue did not decline significantly over time. Univariable analysis identified nicotine use, loss of consciousness at ictus (LOCi), rebleed prior to aneurysm repair, reduced consciousness to Glasgow Coma Scale (GCS) < 14, large amounts of subarachnoid blood, the presence of acute hydrocephalus, and severe vasospasm as factors that were significantly associated with fatigue. In multivariable analysis, nicotine use, reduced GCS, and severe vasospasm were independent predictors that all more than doubled the risk to develop post-aSAH fatigue.

**Conclusions:**

Fatigue is a frequent sequel persisting several years after aSAH. Nicotine use, reduced consciousness at admission, and severe vasospasm are independent predictors of fatigue from the acute phase of aSAH. We propose inflammatory cytokines causing dopamine imbalance to be a common denominator for post-aSAH fatigue and the presently identified predictors.

## Introduction

Fatigue can be characterized by “a feeling of lack of energy, weariness, and aversion to effort” [32]. Currently there is no agreed-upon definition or operationalization of fatigue, partly a result of its complex and multidimensional nature. Fatigue is the most prominent sequel in a cluster of residual symptoms after aneurysmal subarachnoid hemorrhage (aSAH) [34]. Although being highly frequent in the early phase [36, 41], post-aSAH fatigue is also present several years after the ictus [7, 44] and it is a debilitating symptom that has a significant impact upon quality of life and the ability to return to work [38, 43]. Estimates of fatigue prevalence after aSAH vary between 31 and 90 % [28]. This large variability may be related to heterogeneity in study population, timing of assessment after the hemorrhage and methods of assessment for fatigue.

Since fatigue is common in many neurological disorders [9], there may be a range of different underlying neurobiological mechanisms. The pathophysiology of fatigue after ischemic and hemorrhagic stroke remains unclear [11] and the knowledge of aSAH-related factors associated with post-aSAH fatigue is even more limited. Only a small number of studies have examined multiple clinical predictors and their relationship with post-aSAH fatigue [7, 26, 37, 41]. In combination with relative small sample sizes in previous studies, factors associated with post-aSAH fatigue have not been comprehensively evaluated.

## Subjects and methods

### Subjects

The current study was part of the pre-screening for a double-blind, randomized, placebo-controlled study investigating the effect of OSU6162 in the treatment of fatigue and other neuropsychological sequelae after aSAH (http://www.clinicaltrials.gov. Unique identifier: NCT03209830) approved by Health Research Ethics (REC, reference: 2016/2214). Patients (≥ 18 years) that had suffered aSAH at least 1 year earlier and who were treated for their aSAH at our department between January 2012 and December 2017 were contacted. Patients who agreed to participate were interviewed by phone and gave an oral informed consent. Patients living permanently in an institution and those not speaking adequately Norwegian were excluded. The data collection protocol was approved by the institutional review board. The study data that support the findings of this study are available from the corresponding author on reasonable request.

### Measures

#### Fatigue

Fatigue in the chronic phase (≥ 1 year) after aSAH was assessed using the Norwegian version of the Fatigue Severity Scale (FSS) [27, 30] which consists of nine statements about the impact of fatigue on daily life; each statement is scored on a 7-point Likert scale, ranging from 1 (strongly disagree) to 7 (strongly agree). The total FSS score is the mean of the item scores. A mean score of 4 or more is considered outside the range of healthy controls [27] and indicates a moderate to high impact of fatigue on daily living.

#### Predictors

Data on demographics (age and sex), medical condition at admission, and clinical course during the acute phase of aSAH were obtained from our institutional quality registry. We registered nicotine use at the time of ictus, if there was an abrupt LOCi including seizures, rebleed before aneurysm repair, clinical grade just prior to aneurysm repair or prior to intubation in patients admitted intubated (Hunt and Hess Scale [HH] [21] and Glasgow Coma Scale (GCS) [23]), aneurysm location, and the method of aneurysm repair. From the diagnostic computed tomography (CT), we scored the presence of intraparenchymal hemorrhage, the amount of subarachnoid blood (modified Fisher grade [18]), and the amount of intraventricular blood (modified LeRoux score [29], where no intraventricular blood was scored as “0”). Furthermore, we registered if the patient was treated for acute hydrocephalus (need of external drainage of CSF), or for chronic hydrocephalus (implanted shunt). All patients underwent a CT/CT angiography (CTA) on the first day after aneurysm repair and on day 5 in intubated patients and on day 7 in awake patients. Transcranial Doppler ultrasonography (TCD) was performed from day 4 and at regular intervals. We scored the presence of severe vasospasm if CT angiography scans showed a > 50% diameter reduction in one or several vessels and/or TCD showing a Lindegaard ratio [31] > 6. The acquisition of a new cerebral infarction, including those that were procedure-related, was evaluated from CT scans performed during the primary hospital stay or from magnetic resonance imaging (MRI) if performed.

### Statistical analysis

Patient characteristics are presented as mean values with standard deviation (SD) or proportions. Differences in continuous variables between groups were tested using independent sample *t* test or one-way ANOVA. The chi-square test for contingency tables was used to detect associations between categorical variables. To evaluate the frequency of fatigue across time intervals in years since ictus, a Mantel-Haenszel test for trend was performed. Univariable and multivariable logistic regression analyses were performed to identify possible predictors of fatigue (FSS ≥ 4): LOCi (yes or no), rebleed (yes or no), Hunt and Hess scale (1–3 or 4–5), modified Fisher scale (0–2 or 3–4), modified LeRoux score (0–5 or 6–16), severe vasospasm (yes or no), acute hydrocephalus (yes or no), chronic hydrocephalus (yes or no), intracerebral hemorrhage (ICH) (yes or no), and cerebral infarction (yes or no) were dichotomized. Aneurysm location, GCS, and nicotine use were entered by using 3 dummy variables. Any variable with *p* < 0.05 in the univariable analysis was considered a candidate for the multivariable model. Subsequent multivariable logistic regression analyses with manual backward elimination were performed. The associations between potential predictors and fatigue were quantified by odds ratio (OR) with 95% confidence interval (CI). Multivariable analyses were preceded by estimation of correlation between predictors. Two-tailed *p* values of less than 5% were considered statistically significant. All statistical analyses were performed using the IBM SPSS statistics version 25.0 (IBM SPSS Inc., Armonk, NY: IBM Corp).

## Results

**Fig. 1:**
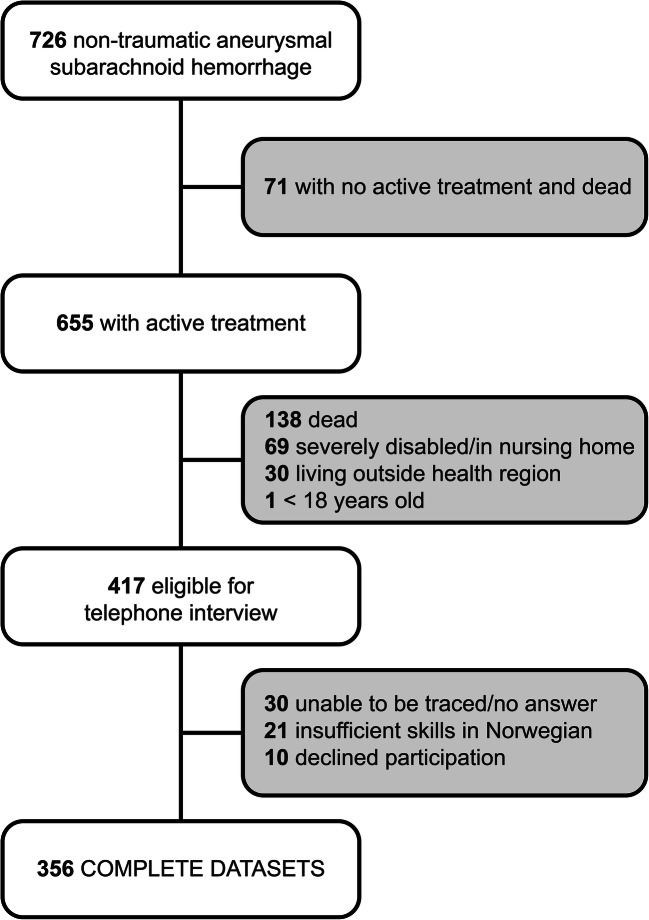
Patient enrollment

A total of 726 patients were admitted for non-traumatic aSAH between January 2012 and December 2017. Figure 1 shows the flow chart of eligible and included patients. A total of 655 patients received active treatment. In this group, 138 died, 69 were severely disabled and/or resided in a nursing home, 30 were living outside Health Region South East, and one was younger than 18 years. Therefore, 417 were eligible for telephone interview, of whom 356 (85%) participated in the study. We excluded 21 patients with insufficient skills in Norwegian, 30 patients were unable to be traced or did not answer, and 10 patients declined participation. The characteristics of the 356 included patients are displayed in Table 1.

**Table 1:** Characteristics of patients with aSAH (*n* = 356)

	*n*	%
Mean age at ictus, years	55.7 ± 12.5	
Sex, male	115	32.3
Predictors		
Aneurysm location		
ACoA/ACA	148	41.6
MCA/ICA	154	43.3
Vertebrobasilar	54	15.2
Treatment		
Spontaneous aneurysm thrombosis	2	0.6
Endovascular	194	54.5
Surgical	160	42.7
Hunt and Hess (HH)		
HH 1–3	284	79.8
HH 4–5	72	20.2
Glasgow Coma Score (GCS)		
GCS 15–14	236	66.3
GCS 13–9	59	16.6
GCS 8–3	61	17.1
Modified Fisher		
0–2	169	47.5
3–4	186	52.2
Modified LeRoux		
0–5	301	84.6
6–16	54	15.2
Nicotine use		
Current	191	53.7
Former	56	15,7
Never	106	29,8
Loss of consciousness at ictus (LOCi)	146	41.0
Rebleed before aneurysm repair	30	8.4
Severe vasospasm	60	16.9
Acute hydrocephalus	239	67.1
Chronic hydrocephalus	83	23.3
Intracerebral hemorrhage	72	20.2
New cerebral infarction	97	27.2
Fatigue		
Mean follow-up time after aSAH in months (SD); range in months	37.6 (23.9); 12–81	
Fatigue Severity Scale (FSS)		
Mean FSS (SD)	4.7 (1.7)	
Clinical fatigue (mean FSS ≥ 4)	248	69.7

### Prevalence and duration of fatigue

**Fig. 2:**
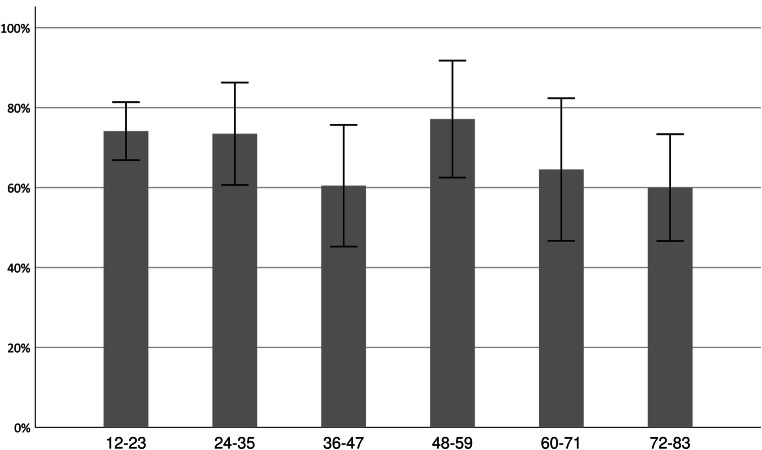
Percentage of patients with fatigue in relation to months passed since ictus

The mean FSS score was 4.7 (SD, 1.7) and fatigue (FSS ≥ 4) was present in 248 patients (69.7%) (Table 1). Figure 2 shows the prevalence of fatigue in relation to time passed since the aSAH. From 1 and up to 7 years after the ictus, fatigue was present in 74%, 74%, 61%, 77%, 65%, and 60%, respectively. Even though there seemed to be a tendency towards decline of fatigue as a function of time, the Mantel-Haenszel test for trend demonstrated that fatigue was stable over time (*p* 0.057).

### Predictors of fatigue

**Fig. 3:**
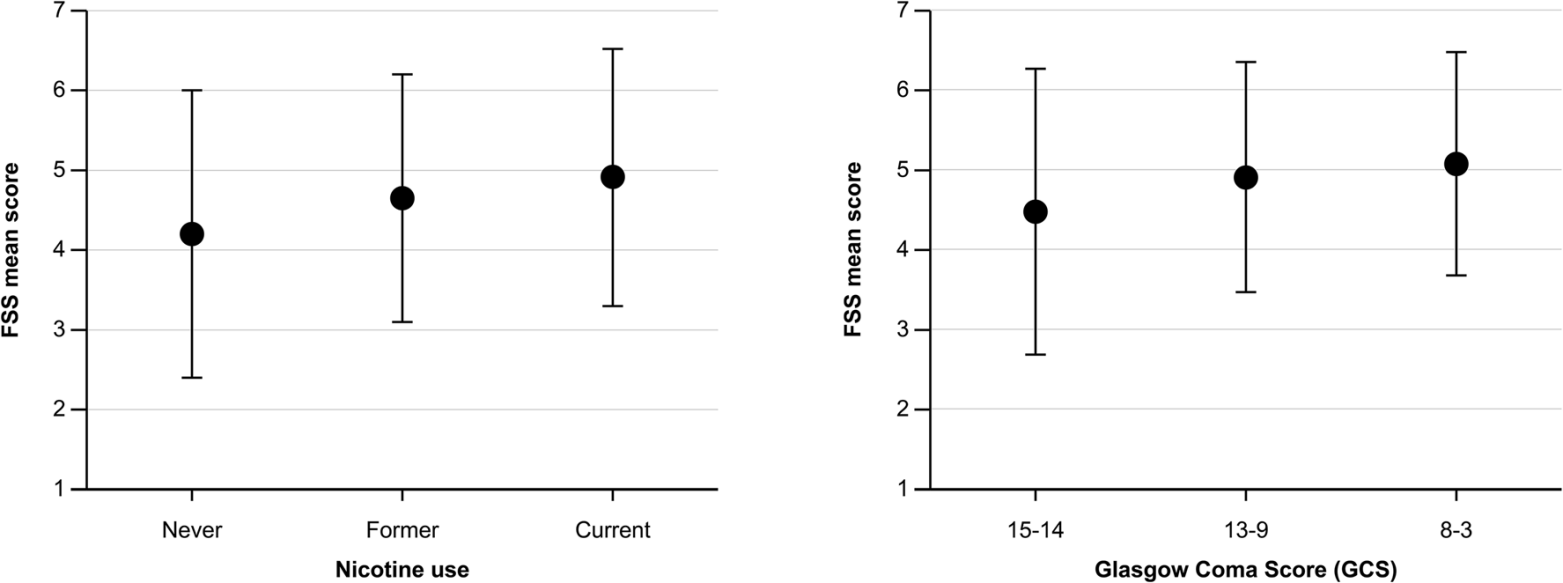
Mean Fatigue Severity Score (FSS) versus nicotine use (left) and Glasgow Coma Score (right)

Results of the univariable analyses on predictors linked to the acute phase of aSAH (demographic, medical, and radiological data) are presented in Table 2. Nicotine use (OR 2.49, 95% CI 1.50–4.14; *p* < 0.001), reduced consciousness to GCS 13–9 (OR 2.46, 95% CI 1.21–4.98; *p* = 0.013) and GCS 8–3 (OR 2.30, 95% CI 1.16–4.56; *p* = 0.017), larger amount of subarachnoid blood (modified Fisher scales 3 and 4: OR 1.86, 95% CI 1.18–2.94; *p* = 0.008), LOCi (OR 2.04, 95% CI 1.26–3.30; *p* = 0.004), rebleed before aneurysm repair (OR 3.05, 95% CI 1.04–8.95; *p* = 0.043), severe vasospasm (OR 2.49, 95% CI 1.21–5.12; *p* = 0.013), and acute hydrocephalus (OR 2.08, 95% CI 1.30–3.32; *p* = 0.002) were identified as statistically significant predictors of fatigue. The mean FSS was significantly lower in never nicotine users (4.20 ± 1.80) than in former users (4.65 ± 1.55) and current users (4.91 ± 1.61) (*p* = 0.002; Fig. 3, left). Likewise, the mean FSS was significantly lower in patients with GCS 15–14 (4.48 ± 1.79) than in those with GCS 13–9 (4.91 ± 1.44) and those with GCS 8–3 (5.08 ± 1.40) (*p* = 0.020; Fig. 3, right).

**Table 2:** Univariable and multivariable analyses of predictors in the study population with and without fatigue (data are presented as the absolute number of patients with percentages in parentheses with the exception of age, which is listed as mean value ± SD)

Variable^†^	Fatigue Severity Scale (FSS)		Univariable analysis		Multivariable analysis	
	FSS ≥ 4 *n* = 248	FSS < 4 *n* = 108	Odds ratio (95% CI)	*p* value	Odds ratio (95% CI)	*p* value
Age in years at ictus, mean ± SD	55.4 ± 11.4	56.5 ± 14.8	0.993 (0.975–1.011)	0.438		
Sex, male	84 (33.9%)	31 (28.7%)	1.272 (0.777–2.083)	0.338		
Nicotine use at time of ictus						
Never	60 (24.4%)	46 (43.0%)	1.000 (ref.)	0.002		
Former	40 (16.3%)	16 (15.0%)	1.917 (0.956–3.842)	0.067		
Current	146 (54.1%)	45 (42.1%)	2.487 (1.495–4.139)	< 0.001	2.104 (1.305–3.394)^‡^	0.002
Aneurysm location						
ACoA/ACA	109 (44.0%)	39 (36.1%)	1.000 (ref.)	0.369		
MCA/ICA	102 (41.1%)	52 (48.1%)	0.702 (0.428–1.152)	0.161		
Vertebrobasilar	37 (14.9%)	17 (15.7%)	0.779 (0.394–1.538)	0.472		
Endovascular treatment	139 (56.3%)	55 (51.4%)	1.217 (0.772–1.918)	0.398		
Hunt and Hess 4–5	57 (23.0%)	15 (13.9%)	1.850 (0.995–3.441)	0.052		
Glasgow Coma Scale						
GCS 15–14	151 (60.9%)	85 (78.7%)	1.000 (ref.)	0.006	1.000 (ref.)	0.011
GCS 13–9	48 (19.4%)	11 (10.2%)	2.456 (1.211–4.981)	0.013	2.490 (1.206–5.140)	0.014
GCS 8–3	49 (19.8%)	12 (11.1%)	2.299 (1.159–4.560)	0.017	2.128 (1.034–4.381)	0.040
Modified Fisher 3–4	141 (57.1%)	45 (41.7%)	1.862 (1.178–2.944)	0.008	1.403 (0.837–2.350)	0.198
Modified LeRoux 6–16	37 (15.0%)	17 (15.7%)	0.943 (0.505–1.762)	0.854		
Loss of consciousness at ictus	114 (46.2%)	32 (29.6%)	2.036 (1.256–3.299)	0.004	1.196 (0.630–2.272)	0.584
Rebleed before treatment	26 (10.5%)	4 (3.7%)	3.045 (1.036–8.950)	0.043	2.680 (0.872–8.236)	0.085
Severe vasospasm	50 (20.2%)	10 (9.3%)	2.487 (1.210–5.115)	0.013	2.298 (1.095–4.823)	0.028
Acute hydrocephalus	179 (72.2%)	60 (55.6%)	2.075 (1.297–3.322	0.002	1.268 (0.712–2.258)	0.419
Chronic hydrocephalus	65 (26.2%)	18 (16.7%)	1.776 (0.995–3.171)	0.052		
Intracerebral hemorrhage	54 (21.8%)	18 (16.7%)	1.392 (0.772–2.508)	0.271		
New cerebral infarction	73 (29.4%)	24 (22.2%)	1.460 (0.860–2.479)	0.161		

^†^Some variables have missing values (number of missing patients in parentheses): nicotine use at time of ictus (3 missing), treatment modality (2 with spontaneous aneurysm thrombosis are excluded), modified Fisher (1 missing), LeRoux (1 missing), LOCi (1 missing), and severe vasospasm (1 missing). ^‡^Nicotine use were dichotomized (never/former versus current)

The final multivariable regression model is presented in Table 2, right column, and identified nicotine use, GCS < 14, and severe vasospasm as independent predictors which approximately doubled the risk to develop fatigue after aSAH: nicotine use (OR 2.10, 95% CI 1.31–3.39; *p* = 0.002) as compared with former and never nicotine use, patients in GCS 13–9 (OR 2.49, 95% CI 1.21–5.14; *p* = 0.014) and GCS 8–3 (OR 2.13, 95% CI 1.03–4.38; *p* = 0.040) as compared with patients in GCS 15–14, and patients with severe vasospasm (OR 2.30, 95% CI 1.10–4.82; *p* = 0.028) as compared with patients with less severe or no vasospasm.

**Fig. 4:**
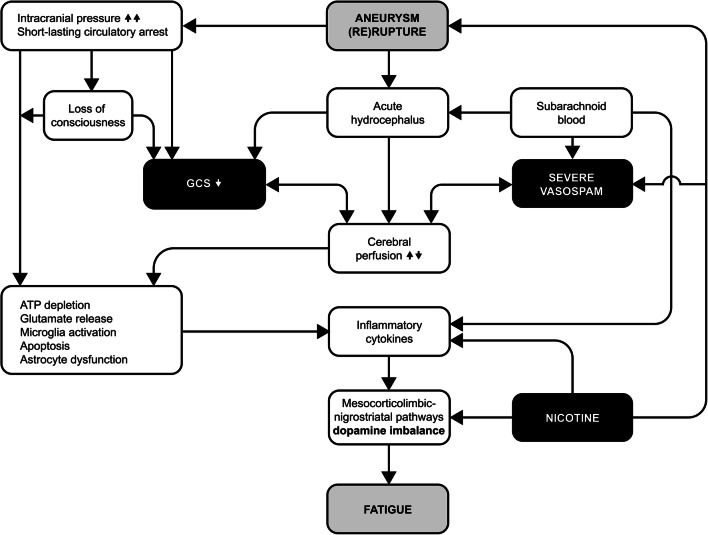
Relationship of variables of aneurysmal hemorrhage, which culminate in processes attributable to the development of fatigue. Black boxes indicate independent predictors of fatigue in the present study. GCS, Glasgow Coma Score [23]; ATP, adenosine triphosphate

The interwoven relationship of clinical variables related to aSAH is illustrated in Fig. 4. We focused on the presently identified predictors of post-aSAH fatigue and how they possibly may culminate in processes leading to or facilitating the development of fatigue.

## Discussion

The core finding in the present study was that the prevalence of fatigue in the chronic phase after aSAH was 69.7% and remained stable over at least 1 to 7 years after the hemorrhage. Nicotine use, reduced consciousness at admission, and severe vasospasm in the acute phase were independent predictors of fatigue.

### Prevalence of post-aSAH fatigue

Our prevalence of 70% is consistent with previous studies that assessed the prevalence of fatigue by means of the FSS questionnaire in patients surviving aSAH [28]. However, other studies that used single questions to assess fatigue reported a much more widespread prevalence [28]. The variance in prevalence is not surprising since there is no “gold standard” or objective measure of fatigue, and the lack of consensus regarding the definition of fatigue is therefore a challenge for allowing consistent measures [13]. Self-report measures of fatigue have major limitations, especially questionnaires that fail to regard fatigue as a multidimensional state with cognitive and emotional/psychological components. Another factor contributing to varying results regarding prevalence of fatigue is low sample size. To the best of our knowledge, the 356 aSAH patients we presently examined comprise by far the largest cohort investigated on this topic to date.

### Timely evolvement of post-aSAH fatigue

Previous longitudinal studies show a consistency in fatigue levels over time [3, 19, 26, 39], where no improvement of fatigue was found in studies with follow-up assessment up to 4 years after the ictus [3, 19]. In line with our findings, other cross-sectional studies [6, 7, 43] have shown a high prevalence of fatigue (47–66%) up to 10 years after the ictus. A systematic review by Kutlubaev et al. [28] reported a higher frequency of fatigue less than 1 year compared with more than 1 year after the ictus (73.6% and 50.7%, respectively), which they postulate can be explained by different mechanisms driving fatigue as a function of time since ictus. On the other hand, in a meta-analysis by Cumming et al. [10], a marked between-study variability in the estimates of fatigue prevalence was not accounted for by timing of assessment after stroke. The authors therefore concluded fatigue to be persistent across time. Hitherto, there are insufficient data to draw any firm conclusions regarding time course; however, there are strong indications that fatigue remains relatively stable after the acute phase. Our data were acquired in patients where the hemorrhage was at least 1 year ago and support the notion that fatigue beyond 1 year is best understood as a chronic condition.

### The dopamine imbalance hypothesis

Not only the definition and quantification of fatigue is challenging, also the underlying biomedical mechanisms remain unclear. Since central fatigue [8] is seen in a wide specter of diseases that share some kind of proinflammatory mechanism, the hypothesis of cytokine-induced inflammation-mediated fatigue has emerged [4, 25, 40]. Inflammatory cytokines have an effect on dopamine release and proper function in the striatum [17]. Such findings support the notion that inflammatory cytokines lead to dopamine imbalance [14] in the dopaminergic pathways, namely the mesocorticolimbic and nigrostriatal systems. The dopamine imbalance hypothesis states that dopamine may play an important role in the perception of fatigue because it is central for cognition, motivation, and effortful behavior [1, 14]. In fact, individuals with high levels of fatigue have reduced mesocorticolimbic connectivity [16]. Dopamine is often referred to as the “reward neurotransmitter,” delicately balancing if a reward is worth the effort [1]. The effect of dopamine follows an inverted “u” shape, with optimal effect on the top of the curve; i.e., fatigue can be caused by too little as well as too much dopamine [14].

### Predictors of post-aSAH fatigue

These hypotheses are relevant in relation to the presently identified predictors of fatigue. Our strongest predictor was use of nicotine. Nicotine addiction is created and maintained in the mesocorticolimbic system where it binds to nicotinic acetylcholine receptors and increases the firing rate and phasic bursts in midbrain dopamine neurons [2]. Chronic nicotine use leads to neuroadaptation and changes in dopamine homeostasis [2], possibly shifting dopamine levels to a point on the inverted “u” curve that promotes the development of fatigue. Nicotine is also a well-established risk factor for aSAH and clinical symptomatic vasospasm and delayed ischemic neurologic deficit in the course of aSAH [12]. Furthermore, nicotine aggravates the post-ischemic inflammatory response and thereby increases brain infarction size [5]. Common background factors for individuals with fatigue and nicotine users, like socio-economic factors and passive coping style, should also be considered.

Despite the large impact of nicotine on the same dopaminergic pathways that seem to be crucial in the development of fatigue, no other studies have investigated this correlation. To our knowledge, merely four previous studies [7, 26, 37, 41] have looked at the relationship between acute SAH-related factors and fatigue in the chronic phase, where three of the studies investigated their patients within 14 months after the hemorrhage. Rödholm et al. [41] found no significant association between fatigue (as defined by astheno-emotional disorder) and reaction level upon admission, amount of subarachnoid blood, or acute hydrocephalus. Passier et al. [37] found no significant association between fatigue assessed with the FSS and clinical status at admission, aneurysm localization, method of aneurysm repair, rebleed, secondary ischemia, or hydrocephalus. Khajeh et al. [26] found severity of SAH (in terms of World Federation of Neurosurgical Societies) to be associated with fatigue, but all other clinical characteristics (i.e., age, gender, body mass index, hydrocephalus, vasospasm, delayed cerebral ischemia (DCI), intraventricular hemorrhage (IVH), intraparenchymal hemorrhage, and rebleed) were not predictive of persistent fatigue. Buunk et al. [7] found a significant relationship between mental fatigue and external CSF drainage, but not with SAH type. Our results confirm in part their findings of lack of relation between clinical variables and fatigue; however, our univariable regression analysis identified GCS, rebleed, acute hydrocephalus, and large amounts of subarachnoid blood as relevant predictors of fatigue. This may be due to differences in evaluating the various variables or that our study cohort is more than three times larger than the patient populations examined in the earlier studies, rendering our results more robust.

Relevant predictors in our study, like LOCi, GCS, large amount of subarachnoid blood, and severe vasospasm, can also be linked to the inflammatory cytokine hypothesis with dopamine imbalance: when an aneurysm ruptures, there is an instant, vast increase in intracranial pressure (ICP), leading to cerebral circulatory arrest of varying length [35]. In some patients, the ICP increase is so brief or so moderate that they do not become unconscious, whereas up to half of aSAH patients experience some form of LOCi [20]. Prolonged LOCi often will lead to the patient being admitted with reduced GCS. If no LOCi occurred, or when awakening from LOCi, acute hydrocephalus can cause an additional and gradual decline in GCS. Presently, acute hydrocephalus was a relevant factor in univariable regression analysis, but did not prove to be an independent predictor like GCS, where we also saw that mean FSS increased the lower GCS was. Even very short-lasting circulatory arrest initiates a cascade of cellular pathophysiologic events as neuronal oxygen stores are depleted within 15–20 s [42]. Prolonged reduced consciousness in the setting of untreated acute aSAH is the result of local parenchymal damage and suboptimal cerebral perfusion pressure (CPP). This results in anaerobic glycolysis in the first 4–5 min and leads to depletion of brain glucose and adenosine triphosphate [33]. Such inflammation and downregulation of astrocytes leading to dysfunctional glutamate transmission have been attributed to mental fatigue after traumatic brain injury [24]. Microglia and endothelial cells become activated, and through complex mechanisms, proinflammatory cytokines (among others tumor necrosis factor-α, IL-6, and IL-8) are released [33].

The most feared complication during the first 2 weeks after aSAH is cerebral vasospasm [22]. Most patients experience some degree of vasospasm in conjunction with their aSAH, whereas usually only severe vasospasm becomes symptomatic due to reduced CPP. Without treatment adjustments to preserve adequate CPP, cerebral ischemia can occur or be aggravated [22]. Large amounts of subarachnoid blood represent a risk factor for developing vasospasm [22]. We found severe vasospasm to be an independent predictor of fatigue and large amount of subarachnoid blood was presently a predictor in the univariable analysis. Vasospasm after aSAH is not a purely mechanical event, but develops over days as an inflammatory response to blood degradation [15] and leads to a thickening of the arterial wall and thereby reduced arterial lumen. The inflammatory cytokines found in vasospasm comprise tumor necrosis factor-α, IL-1α, IL-1β, IL-6, and IL-8 [15], and in severe vasospasm, IL-6 seems to be predominant [22]. Hence, vasospasm is linked to the same inflammatory cytokines that are upregulated in patients with ischemia and/or reduced consciousness. Of these, IL-6 and tumor necrosis factor-α have also been directly related to the development of fatigue [40]. Inhibitors of tumor necrosis factor-α and antibodies against IL-1β have improved fatigue symptoms in patients with psoriasis, diabetes, and rheumatoid arthritis [25]. Substances ameliorating the dopamine imbalance, like methylphenidate or OSU6162, have also been mentioned as potential pharmacological therapies of fatigue [24]. Figure 4 illustrates the complex, interwoven relationship of the presently investigated variables of aSAH, which eventually may culminate in processes attributable to the development of fatigue.

## Limitations

This study has several limitations. We used only one measure of fatigue, and there may have been other aspects of fatigue that remained under- or overestimated by the use of the FSS questionnaire. It is also uncertain whether administration of FSS by telephone interview in comparison with standard paper-and-pencil self-administration influenced the patients’ response style. We showed the frequency of fatigue to be remarkable stable up to 7 years after ictus, but time trajectories cannot be established with our cross-sectional study design. Although common to many stroke studies, generalizability of our findings was limited by excluding patients living at a nursing home, with pronounced cognitive sequelae or aphasia. This concerned only a small group of survivors (< 15%); however, it might have led to a selection bias where our estimate is an inaccurate reflection of fatigue prevalence in the overall aSAH population. On the other hand, we included patients from the entire range of aSAH severity. Finally, we assessed factors related to clinical status in the acute phase. Several other predictors such as premorbid personality traits (e.g., neuroticism, coping style), psychiatric comorbidity (e.g., mood disorder), and somatic comorbidity (e.g., sleep disorder, hypopituitarism, cancer, rheumatic diseases) were not measured. Future studies should include an even bigger range of possible factors related to fatigue. Altogether, these limitations should be considered when interpreting our findings. Still, our study included a large number of patients and is novel in that it could identify relevant clinical predictors of post-aSAH fatigue.

## Conclusions

The prevalence of fatigue in the chronic phase after aSAH was 69.7%. Fatigue remained a common and stable symptom up to 7 years after the ictus. Nicotine use, reduced consciousness with GCS < 14 at admission, and severe vasospasm were independent predictors from the acute phase of aSAH that more than doubled the risk to develop post-aSAH fatigue. Inflammatory cytokines causing dopamine imbalance may be a common denominator for fatigue and the presently identified predictors.

## Abbreviations

ACA: Anterior cerebral artery
ACoA: Anterior communicating artery
aSAH: Aneurysmal subarachnoid hemorrhage
ATP: Adenosine triphosphate
CT: Computed tomography
CTA: Computed tomography angiography
CPP: Cerebral perfusion pressure
CSF: Cerebrospinal fluid
DCI: Delayed cerebral ischemia
FSS: Fatigue Severity Scale
GCS: Glasgow Coma Scale
HH: Hunt and Hess Scale
ICA: Internal carotid artery
ICH: Intracerebral hemorrhage
IVH: Intraventricular hemorrhage
ICP: Intracranial pressure
LOCi: Loss of consciousness at ictus
MCA: Middle cerebral artery
MRI: Magnetic resonance imaging
PTSD: Post-traumatic stress disorder
TCD: Transcranial Doppler ultrasonography
